# Identification of Gene Variants Associated with Melanocyte Stem Cell Differentiation in Mice Predisposed for Hair Graying

**DOI:** 10.1534/g3.118.200965

**Published:** 2019-01-16

**Authors:** Allison C. Fialkowski, Denise J. Levy, Dawn E. Watkins-Chow, Joseph W. Palmer, Roshan Darji, Hemant K. Tiwari, William J. Pavan, Melissa L. Harris

**Affiliations:** * Department of Biostatistics; ‡ University of Alabama at Birmingham, Birmingham, AL, and; † Genetic Disease Research Branch, National Human Genome Research Institute, National Institutes of Health, Bethesda, MD

**Keywords:** hair graying, melanocyte stem cell, QTL, modifier

## Abstract

Age-related hair graying is caused by malfunction in the regenerative potential of the adult pigmentation system. The retention of hair color over the life of an organism is dependent on the ability of the melanocyte stem cells and their progeny to produce pigment each time a new hair grows. Age-related hair graying is variable in association with genetic background suggesting that quantitative trait loci influencing this trait can be identified. Identification of these quantitative trait loci may lead to the discovery of novel and interesting genes involved in stem cell biology and/or melanogenesis. With this in mind we developed previously a sensitized, mouse modifier screen and discovered that the DBA/1J background is particularly resistant to melanocyte stem cell differentiation in comparison to the C57BL/6J background. Melanocyte stem cell differentiation generally precedes hair graying and is observed in melanocyte stem cells with age. Using quantitative trait loci analysis, we have now identified three quantitative trait loci on mouse chromosomes 7, 13, and X that are associated with DBA/1J-mediated variability in melanocyte stem cell differentiation. Taking advantage of publicly-available mouse sequence and variant data, in silico protein prediction programs, and whole genome gene expression results we describe a short list of potential candidate genes that we anticipate to be involved in melanocyte stem cell biology in mice.

In humans, the visible phenotype of graying scalp hair is associated primarily with aging and varies among people of different ethnic or ancestral geographic origin. In people 45-65 years of age, the intensity (percent per individual) and frequency (percent of individuals) of gray hair ranges from 15 to 42% and 43–93%, respectively (Panhard *et al.* 2012). The fact that these differences are associated with ancestry, or genetic background, suggest that hair graying is, in part, a genetic trait. The quantitative nature of hair graying further suggests that this trait is influenced by the combination of effects at multiple genetic loci. Gray hair is easy to dismiss as a purely cosmetic phenotype, however, research into the molecular etiology of this phenotype has contributed to our basic understanding of stem cell biology, tissue regeneration and homeostasis, melanocyte-related diseases like vitiligo and melanoma, and the role of stem cells in aging phenotypes. Accordingly, we are interested in identifying the genetic modifications that contribute to hair graying variation in the hopes of discovering novel genes that participate in these processes.

Unfortunately, genetic studies of gray hair in humans can be difficult to perform due to the confounding variable associated with hair dyeing. Accurate phenotyping can only be performed in individuals and regions of the scalp (near the roots) that maintain natural hair color. Not surprisingly, to date there is only one reported genome-wide association study that has successfully identified a genetic locus, *IRF4*, that is involved in age-related hair graying in humans (Adhikari *et al.* 2016). As an alternate to studying pigmentation in humans, the mouse model system has long been used to investigate genetic and molecular mechanisms related to melanocyte biology (Jackson 1994, 1997; Steingrímsson *et al.* 2006). Thus, we sought to assess whether mice, as an alternate to humans, could help in the identification of genetic variants that contribute the phenotypic diversity of hair graying. Previously we reported the development of a sensitized screen to evaluate genetically diverse inbred mouse strains for their ability to influence hair graying (Harris *et al.* 2015). In this screen we employed the Tg(DctSox10) transgenic mouse line to predispose mice to hair graying. The cellular mechanism responsible for hair graying in these mice is melanocyte stem cell (McSC) differentiation. This phenomenon precedes hair graying and is positively associated with hair graying severity (Harris *et al.* 2013). Differentiated McSCs have also been observed in human hairs and their number increases with age (Nishimura *et al.* 2005), which makes McSC differentation a relevant cellular phenotype to evaluate for genetic loci that may modify the extent of age-related hair graying in both mouse and humans. Mechanistically we predict that modifier genes that effect McSC differentiation may directly regulate the process of melanogenesis, but could also be involved in initial McSC establishment, proliferation or migration of McSC progenitors.

McSCs that are undergoing differentiation produce visible ectopic pigmentation when viewed by light microscopy and the number of hairs that contain these ectopically pigmented McSCs (EPMs) varies in animals of different genetic backgrounds (Harris *et al.* 2015). Mice that are hemizygous for a conditional, *Sox10*-expressing transgene, designated C57BL/6J-Tg(DctSox10)/0, are extremely susceptible to McSC differentiation. In contrast, progeny derived from mating C57BL/6J-Tg(DctSox10)/0 mice to other inbred genetic backgrounds (C3H/HeJ, 129SvEvTac, FVB/NTac, DBA/1J, BALB/CJ) produced F1 hybrids that all exhibit reduced numbers of hairs with EPMs in response to the transgene (Harris *et al.* 2015). Tg(DctSox10)/0 hybrid animals produced by mating C57BL/6J-Tg(DctSox10)/0 mice to DBA/1J mice exhibit a particularly low level of transgene-mediated EPMs. Reduction of EPMs in these F1 hybrids suggests a dominant mode of inheritance for EPM resistance and we sought to identify these DBA/1J-associated resistance loci using QTL linkage mapping.

## Methods

### Mice

C57BL/6J and DBA/1J mice were obtained from the Jackson Laboratory. The Tg(Dct-Sox10) transgenic line Tg(Dct-Sox10)CF1-10Pav (Hakami *et al.* 2006)) was generated previously, established on the FVB/N background and maintained through a combination of backcrossing to C57BL/6J and by intercross. All (DBA/1J x C57BL/6J-Tg(DctSox10)/0)F1-Tg(DctSox10)/0 animals (abbreviated as D1B6F1-Tg(DctSox10)/0) used in this study were generated by mating one C57BL/6J-Tg(DctSox10)/0 male to several DBA/1J females. D1B6F1-Tg(DctSox10)/0 females were then mated to individual C57BL/6J males to generate N2 backcross progeny.

### Phenotype Analysis

Assessment of hairs with EPMs was performed as described previously (Harris *et al.* 2015). Briefly, between 9-11 weeks of age, hair along a 2x2 cm region of the lower back was plucked by hand to synchronize and initiate hair regrowth. Hairs in the plucked region were allowed to regrow for seven days (equivalent to hair cycle stage anagen III/IV). Skin from these animals was dissected and processed for cryosectioning. Using light microscopy, approximately four 10 μm sections were analyzed in total skipping at least three sections between those analyzed to prevent counting the same hair twice. EPMs occur in the hair bulge at the insertion point of the arrector pili muscle, thus only sectioned hair follicles that spanned the entire region from the sebaceous gland and past the junction of the dermal/subcutis were counted. Between 50-100 hairs were examined for the presence or absence of EPMs. The percentage of hairs with EPMs was calculated by dividing the number of hairs with EPMs by the total number of hairs analyzed. 122 N2 mice were phenotyped. For QTL analysis, selective genotyping was performed on 79 of the N2 mice exhibiting the highest (> 50%, n = 39) and lowest (<20%, n = 40) percentage of hairs exhibiting EPMs. The EPM phenotype was converted to a binary trait with the high EPM phenotype scored as 1 and referred to as ‘affected’, while the low EPM phenotype was scored as 0 and referred to as ‘unaffected’. Graphing was performed using Graphpad Prism (Graphpad Software). Brightfield microscopy was performed on a Zeiss Observer.D1 compound microscope. Images were obtained with an Axiocam Hrc camera (Zeiss) using the ZEN software (Zeiss) and processed with Adobe Photoshop (Adobe).

### Genotyping

Presence of the Tg(Dct-Sox10) transgene was determined by PCR using primers that generate an amplicon spanning the *Dct* promoter and the *Sox10* cDNA: 5′-AGCAGTATGGCTGGAGCACT-3′; 5′-TCCAGTCGTAGCCGCTGAGCA-3′. PCR cycling was performed as published previously (Harris *et al.* 2013). SNP genotyping was performed on a custom panel of 1449 SNPs (equivalent to the Mouse Medium Density Linkage Panel, Illumina) using the GoldenGate Genotyping Universal-32 Assay Kit with UDG (Illumina). Complete SNP genotyping data are available in Supplemental File 1 (sheet name- Original Sample Genotypes). In preparation for QTL analysis using R/qtl, 559 non-informative SNPs and SNPs with a high number of no call (NC) values or were omitted. Sample genotypes were also recoded such that homozygote genotypes matching the parental C57BL/6J genotype were designated AA, and heterozygote genotypes were designated AB. SNPs with unknown chromosomal coordinates in the original genotyping data (listed as chr 0) were identified in the genome. The chromosomal coordinates of each SNP were then converted to Sex-Averaged cM-G2F1 centimorgan positions using Mouse Map Converter (http://cgd.jax.org/mousemapconverter/). The final data matrix used for QTL analysis is included in Supplemental File 1 (sheet name- Converted Sample Genotypes).

### Statistical analysis

A total of 79 mice were initially evaluated, and 3 removed for low-quality genotyping results. QTL linkage analysis of 76 mice (36 with the high and 40 with the low EPM phenotype, 37 males and 39 females) and 890 markers using EPM as a binary trait was performed using the R/qtl software (Rv.3.4.4, qtl v.1.42-8). One-dimensional scans were conducted without and with sex as an additive covariate using logistic regression with the EM algorithm (Dempster *et al.* 1976; Lander and Botstein 1989; Lander and Botstein 1989). Separate LOD significance thresholds were obtained from 1,000 permutations for autosomal SNPs and 18,850 permutations for X chromosome SNPs (see R/QTL documentation for explanation of the permutation counts applied within the scanone function). Two-dimensional scans were conducted with sex as an additive covariate using logistic regression with the EM algorithm. Separate LOD significance thresholds were obtained from 1,000 permutations for autosomal SNP pairs, 355,289 permutations for X chromosome SNP pairs, and 9,425 permutations for autosomal:X chromosome SNP pairs (see R/QTL documentation for explanation of the permutation counts applied within the scantwo function). For the QTL found significant at the 0.05 level in the two-QTL analyses, multiple-QTL analyses were performed with sex as an additive covariate using logistic regression with multiple imputation (Sen and Churchill 2001). The locations of the QTL were updated based on maximum likelihood (Zeng *et al.* 1999) using the refineqtl function within R/QTL.

### Identification of candidate genes

Whole skin RNAseq data comparing C57BL/6J to DBA/1J was retrieved at NCBI GEO using the accession # GSE86315. This dataset included read counts previously generated by aligning RNAseq reads to the mouse genome (GRCm38/mm10) using TopHat2, assessment of mapping quality using RSeQC and RNA-SeQC, and read counting using HTSeq (Swindell *et al.* 2017). HTSeq reads from the C57BL/6J and DBA/1J control skin samples (2 males and 2 females per strain all treated with a non-toxic lanolin-derived occlusion cream) were used to generate normalized read counts (median ratio method) and compared to obtain differential expression values using DESeq2. The complete differential expression data including base mean (mean of the normalized counts), log2 fold change, and adjusted p values is provided in Supplemental File 2. Wildtype C57BL/6J McSC RNAseq data were retrieved at NCBI GEO using the accession # GSE102271. RNAseq reads were aligned to the Ensembl GRCm38.p5 primary DNA assembly using STAR (v2.5.2b) and normalized read counts (median ratio method) determined using DESeq2. Normalized read counts with a value of 0 were omitted from further analysis. The complete McSC expression data are provided in Supplemental File 3. Variants between C57BL/6J and DBA/1J were obtained from the Mouse Genome Project (REL-1505; ftp://ftp-mouse.sanger.ac.uk/). The ‘genome variants’ function of PROVEAN (v1.1.3 and GRCm38 Ensembl 74; http://provean.jcvi.org) was used to score variants based on predicted protein function. PROVEAN summary and detailed results are provided in Supplemental File 4.

### Reagent and Data Availability

All data associated with this manuscript is available within the manuscript or as supplemental files. Supplemental Files 1-4 are available via the GSA Figshare portal. Supplemental File 1 provides the SNP genotyping data of the N2 animals. Supplemental File 2 provides the differential mRNA expression data comparing DBA/1J and C57BL/6J skin from the reanalysis of GSE86315. Supplemental File 3 provides the mRNA expression data of wildtype C57BL/6J McSCs from the reanalysis of GSE102271. Supplemental File 4 provides the summary and detailed output from PROVEAN. Supplemental material available at Figshare: https://doi.org/10.25387/g3.7489625.

## Results

### Distribution of EPM susceptibility in progeny derived from backcrossing D1B6F1-Tg(DctSox10)/0 to C57BL/6J

**Figure 1:**
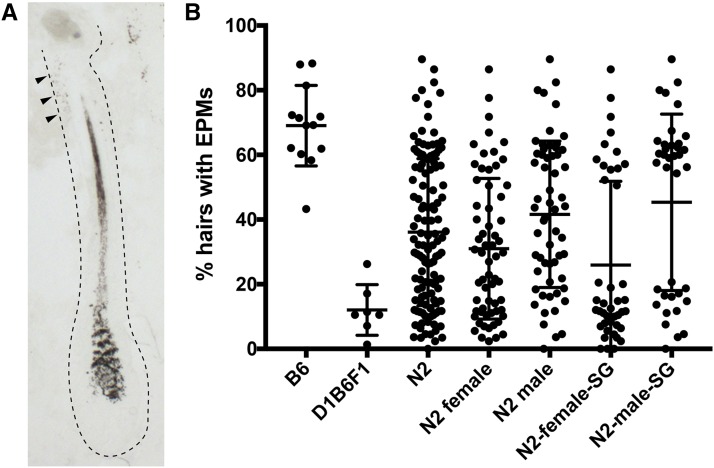
Distribution of hairs with EPMs in N2 mice suggest a quantitative trait. (A) Brightfield image of a histological section of a hair exhibiting EPMs within the stem cell compartment of the hair follicle (arrowheads). Dotted line indicates the position of the hair follicle. (B) Distribution of EPMs in the parental lines (B6 and D1B6F1) and their N2 progeny. N2 progeny are shown together (column 3), separated by sex (column 4 and 5), and only those included for genotyping also separated by sex (column 6 and 7). EPMs, ectopically pigmented melanocyte stem cells; B6, C57BL/6J-Tg(DctSox10)/0; D1B6F1, (DBA/1J x C57BL/6J-Tg(DctSox10)/0)F1-Tg(DctSox10)/0; SG, selective genotyping.

To identify the genetic determinants from DBA/1J that promote EPM resistance we set out to map loci that modify the production of EPMs in progeny produced from backcrossing D1B6F1-Tg(DctSox10)/0 females to C57BL/6J males (these progeny are hereafter referred to as N2 mice). At approximately eight weeks of age, the hair was plucked along the lower back of N2 mice to induce and synchronize hair growth. One week later, skin from these mice was obtained from the plucked region and assessed for EPMs using histological methods (Figure 1a). 122 N2 mice that carry the Tg(DctSox10) transgene were evaluated phenotypically, and exhibited a range of EPM measurements extending between the C57BL/6J-Tg(DctSox10)/0 and D1B6F1-Tg(DctSox10)/0 parental phenotypes (Figure 1b). A statistically significant gender effect (*t*-test, p-value = 0.009) is also observed in N2 mice with the phenotypic mean of the female N2 animals skewed toward more resistant to EPMs suggesting the need for including sex as covariate during QTL mapping.

### QTL analysis provides evidence for three QTL loci associated EPM variability

To map genetic modifiers that affect resistance to McSC differentiation, we used a selective genotyping approach where only the animals with the most extreme phenotypes are genotyped for linkage analysis (based on (Lander and Botstein 1989)). A total of 79 N2 mice were genotyped and represent those animals with the highest (> 50%, n = 39) and lowest (<20%, n = 40) percentage of hairs exhibiting EPMs (Figure 1b). These mice were genotyped using a panel of 1449, evenly-dispersed, mouse-specific SNP loci assays (Illumina, Supplemental File 1). Among the 1449 SNPs evaluated, 890 were found to be reliable and informative between C57BL/6J and DBA/1J. 3 of the original 79 mice had low-quality genotyping scores and were removed prior to QTL analysis.

**Figure 2:**
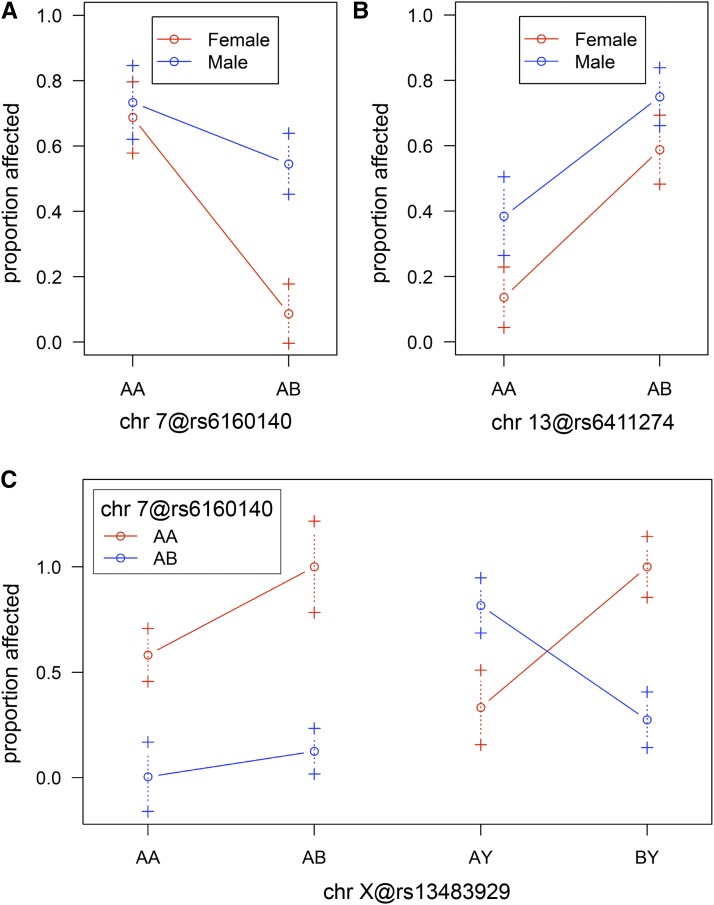
Effect graphs of QTL on chr 7, 13 and the X chromosome. Proportion affected refers to the proportion of animals exhibiting the affected, high EPM binary phenotype. ‘A’ corresponds to the C57BL/6J allele and ‘B’ corresponds to the DBA/1J allele. The genotype is provided at the SNP marker nearest the linkage position with the highest LOD score. (A, B) Proportion of affected individuals as a function of genotype at QTL loci on chr 7 (A) or chr 13 (B). Males and females are represented in blue and red, respectively. (C) Proportion of affected individuals as a function of genotype considering two QTL loci simultaneously. The QTL at chr 7 is indicated by the red and blue lines and the X chromosome QTL is represented on the (x-axis). AA and AB represent the females and AY and BY represent the males. chr, chromosome.

Identification of individual QTL was first performed using a single-QTL genome scan approach (R/qtl; Broman *et al.* 2003). Only the genotyped animals mentioned above were included in these analyses and thus the high and low EPM percentage were treated as a binary trait. Results from the single-QTL analysis, without sex as a covariate and a 5% significance threshold, indicates the presence of one QTL on chr 13 (lod- 3.56, p-value- 0.02; Table 1). Using a 10% significance threshold, there is also weak support for an additional QTL on chr 7 when sex is included as an additive covariate (lod.add- 2.91, p-value- 0.09; Table 1). Seeing as there is a sex-dependent difference in the phenotypic mean of the N2 animals (Figure 1b) we also evaluated for an interaction between QTL and sex and find that the LOD score for the chr 7 QTL increases if an interaction is allowed (lod.full- 3.81, p-value 0.07; Table 1). Stratifying the data by analyzing males and females separately suggests that the chr 7 QTL is female-specific (lod.male- 0.39, p-value- 1.00; lod.female- 3.54, p-value- 0.03; Table 2), however, the interaction between sex and the chr 7 QTL is not significant (lod.int- 0.90, p-value- 0.40). The effects of each QTL were visualized by plotting the proportion affected as a function of genotype at the SNP markers nearest the chromosomal location with the highest LOD score (Figure 2). The effects at the chr 7 QTL are consistent with the DBA/1J allele conferring resistance to McSC differentiation with a low proportion of animals exhibiting the affected, high EPM phenotype in association with heterozygosity (*AB*) for the C57BL/6J and DBA/1J alleles (Figure 2a). In addition, females exhibit a noticeably larger effect than males, matching the above evidence suggesting that the chr 7 QTL is influenced by sex. The chr 13 QTL, on the other hand, exhibits an effect that suggests that this QTL may be involved in DBA/1J-mediated susceptibility to McSC differentiation. In this case, a high proportion of animals exhibit the affected, high EPM phenotype in association with heterozygosity for the C57BL/6J and DBA/1J alleles (Figure 2b). Single-QTL analysis for the chr X was also performed and no loci were identified that met the 10% significance threshold (results not shown).

**Table 1:** Autosomal single QTL analysis for QTL loci linked to EPM variability^1^

all animals without sex as a covariate (lod)					all animals with sex as an additive covariate (lod.add)				
	chr	pos	lod	pval		chr	pos	lod	pval
rs6312657	1	30.99	0.60	1.00	rs6312657	1	30.99	0.48	1.00
CEL-2_168586738	2	79.43	1.42	0.93	rs6376291	2	69.07	1.53	0.84
rs13477487	3	68.08	1.63	0.81	rs13477487	3	68.08	2.40	0.25
rs3659791	4	38.57	1.37	0.95	rs3659791	4	38.57	1.57	0.81
rs3664617	5	0.16	0.45	1.00	c5.loc23	5	23.15	0.51	1.00
rs6389420	6	57.63	1.46	0.91	mhcCD8b4	6	28.46	1.38	0.92
rs6160140	7	28.39	2.60	0.18	**rs6160140**	**7**	**28.39**	**2.91**	**0.09**
rs3656875	8	29.32	2.01	0.47	c8.loc41	8	44.68	2.33	0.28
rs13480345	9	42.94	1.70	0.73	rs13480345	9	42.94	1.27	0.97
rs3699409	10	3.37	1.29	0.97	rs3699409	10	3.37	1.14	1.00
c11.loc15	11	18.55	2.40	0.25	c11.loc15	11	18.55	2.78	0.11
rs3706330	12	6.60	1.12	1.00	rs3706330	12	6.60	1.32	0.95
**c13.loc20**	**13**	**21.79**	**3.56**	**0.02**	**c13.loc20**	**13**	**21.79**	**3.08**	**0.06**
rs6156908	14	31.24	0.98	1.00	rs6156908	14	31.24	0.93	1.00
rs3711814	15	3.58	0.46	1.00	rs3711814	15	3.58	0.46	1.00
c16.loc38	16	39.39	2.36	0.27	c16.loc38	16	39.39	2.19	0.37
c17.loc3	17	3.00	0.28	1.00	rs3706382	17	57.03	0.29	1.00
rs13483244	18	6.53	0.30	1.00	rs6358426	18	5.46	0.40	1.00
rs6236348	19	0.68	0.40	1.00	rs6293693	19	14.23	0.48	1.00
all animals with sex as an interactive covariate (lod.full)									
	chr	pos	lod	pval					
rs6288543	1	30.68	1.14	1.00					
CEL-2_168586738	2	79.43	1.65	0.99					
rs13477487	3	68.08	2.45	0.67					
rs3659791	4	38.57	1.82	0.98					
c5.loc23	5	23.15	0.54	1.00					
rs6389420	6	57.63	1.43	1.00					
**rs6160140**	**7**	**28.39**	**3.81**	**0.07**					
c8.loc41	8	44.68	2.83	0.41					
c9.loc57	9	57.03	2.24	0.82					
CEL-10_58149652	10	23.51	3.15	0.24					
c11.loc15	11	18.55	2.83	0.41					
rs3706330	12	6.60	1.91	0.96					
c13.loc20	13	21.79	3.14	0.25					
rs6156908	14	31.24	0.94	1.00					
CEL-15_8331158	15	1.67	1.23	1.00					
c16.loc38	16	39.39	2.20	0.84					
c17.loc18	17	18.00	0.62	1.00					
rs13483244	18	6.53	1.35	1.00					
rs6236348	19	0.68	1.50	1.00					

1 Results from the single-QTL analysis. Cells shaded in gray highlight loci described in the Results. chr, chromosome; pos, centimorgan position; lod, LOD value; pval, p-value.

**Table 2:** Sex-stratified single-QTL analysis for QTL loci linked to EPM variability^2^

males without sex as a covariate (lod.male)					females without sex as a covariate (lod.female)				
position	chr	pos	lod	pval	position	chr	pos	lod	pval
rs6312657	1	30.99	1.13	0.99	rs13475706	1	0.48	0.59	1.00
CEL-2_135876979	2	60.99	0.94	1.00	rs3726974	2	80.78	1.40	0.94
rs13477487	3	68.08	1.51	0.86	c3.loc44	3	44.22	1.20	0.98
rs3659791	4	38.57	1.56	0.78	rs13477643	4	14.92	0.42	1.00
mCV23386455	5	27.75	0.33	1.00	rs3660964	5	23.12	0.27	1.00
rs6389420	6	57.63	1.15	0.98	rs6238771	6	19.90	1.12	0.99
rs3700068	7	0.26	0.39	1.00	**c7.loc29**	**7**	**29.26**	**3.54**	**0.03**
CEL-8_33812776	8	16.34	1.31	0.95	c8.loc44	8	47.68	2.55	0.20
rs13480271	9	35.75	1.04	1.00	c9.loc57	9	57.03	2.23	0.40
CEL-10_58149652	10	23.51	0.46	1.00	CEL-10_58149652	10	23.51	2.69	0.15
rs6197743	11	38.94	1.98	0.44	c11.loc15	11	18.55	1.77	0.68
rs3706330	12	6.60	1.81	0.62	rs3023711	12	59.74	0.28	1.00
rs6288319	13	40.58	2.29	0.27	c13.loc19	13	20.79	1.98	0.55
gnf14.055.608	14	23.52	0.39	1.00	c14.loc33	14	31.87	0.58	1.00
rs3715857	15	4.87	0.95	1.00	rs6188239	15	10.26	0.33	1.00
rs4201998	16	35.46	1.52	0.80	c16.loc36	16	37.39	1.09	0.99
rs3706382	17	57.03	0.43	1.00	c17.loc3	17	3.00	0.30	1.00
rs13483244	18	6.53	1.27	0.96	c18.loc25	18	24.93	0.47	1.00
rs6236348	19	0.68	1.31	0.93	rs13483525	19	3.11	0.19	1.00

2 Results from the single-QTL analysis stratified by sex. Cells shaded in gray highlight loci described in the Results. chr, chromosome; pos, centimorgan position; lod, LOD value; pval, p-value.

In order to look for additional QTL that may contribute to DBA/1J-mediated EPM resistance, a two-QTL genome scan approach was performed. Results from the two-QTL analyses focusing on pairs of autosomal QTL (with or without sex as an additive covariate) does not identify any pairs of that significantly improve the two-QTL model (lod.full or lod.add) above that of a single QTL (lod.fv1 or lod.av1) when using a 5% significance threshold (Table 3). However, when assessing for pairs of QTL between the autosomes and the X chromosome (with sex as an additive covariate), there is significant evidence for a pair on chr 7 and the X chromosome (Table 3). The full model (lod.full) containing QTL at chr 7 and the X chromosome provides a better fit to the data than both the best single-QTL model (lod.fv1) and the additive model (lod.add). Interaction between these QTL is also significant (lod.int) suggesting that these QTL are epistasic. When the chr 7 and the X chromosome genotypes are considered together (Figure 2c), the effect of the chr 7 QTL in females is the same; a low proportion of individuals exhibit the affected, high EPM phenotype when heterozygous for the chr 7 QTL, independent of genotype at the X chromosome QTL. In males, however, the effect of the chr 7 QTL is opposite depending on whether the X chromosome is C57BL/6J-derived (A allele) or DBA/1J-derived (B allele). Specifically, the effect of the chr 7 QTL switches from EPM resistance in combination with the X chromosome B allele to EPM susceptibility in males with a the X chromosome A allele. No pairs of QTL on the X chromosome approach the criteria for significance (results not shown).

**Table 3:** Two-QTL analysis identifies additional QTL loci linked to EPM variability^3^

Autosome:autosome QTL pairs without sex as an additive covariate									
Full model									
chr1	chr2	pos1f	pos2f	lod.full	*p*	lod.fv1	*p*	lod.int	*p*
7	13	28.255	21.790	6.085	0.090	2.527	1.000	0.063	1.000
8	9	29.681	38.027	4.995	0.496	3.103	0.943	0.102	1.000
9	11	42.027	39.553	5.814	0.150	3.445	0.780	1.014	1.000
Additive model									
chr1	chr2	pos1a	pos2a	lod.add	*p*	lod.av1	*p*		
7	13	28.255	21.790	6.022	0.006	2.464	0.143		
8	9	29.681	56.027	4.893	0.076	3.001	0.028		
9	11	42.027	39.553	4.800	0.091	2.430	0.164		
Autosome:autosome QTL pairs with sex as an additive covariate									
Full model									
chr1	chr2	pos1f	pos2f	lod.full	*p*	lod.fv1	*p*	lod.int	*p*
3	7	68.219	28.255	5.373	0.337	2.527	1.000	0.042	1.000
3	8	68.219	43.681	5.639	0.233	3.257	0.919	0.757	1.000
7	13	28.255	21.790	5.834	0.167	2.753	0.999	0.001	1.000
Additive model									
chr1	chr2	pos1a	pos2a	lod.add	*p*	lod.av1	*p*		
3	7	68.219	28.255	5.331	0.036	2.485	0.158		
3	8	68.219	53.681	4.882	0.096	2.501	0.154		
7	13	28.255	21.790	5.833	0.011	2.752	0.075		
Autosome:X QTL pairs with sex as an additive covariate									
Full model									
chr1	chr2	pos1f	pos2f	lod.full	pval	lod.fv1	pval.1	lod.int	pval.2
7	X	28.255	46.169	8.472	0.004	5.626	0.044	5.143	0.038
Additive model									
chr1	chr2	pos1a	pos2a	lod.add	pval	lod.av1	pval.1		
7	X	28.255	64.169	3.329	0.414	0.483	0.992		

3 Results from the two-QTL analysis. The LOD scores and associated p values (p) are as follows: lod.full- max LOD score for the full model for the chromosome pair; lod.fv1- difference between the full LOD and the max single-QTL LOD for the chromosome pair; lod.add- max LOD score for the additive model for the chromosome pair; lod.av1- difference between the additive LOD and the max single-QTL LOD for the chromosome pair; lod.int- difference between the max full LOD and the max additive LOD for the chromosome pair (taken from r/qtl package documentation; see Broman *et al.*, 2003 for additional details). Cells shaded in gray highlight models described in the Results. chr, chromosome; pos, centimorgan position.

In summary, single-QTL and two-QTL analysis of the genotype and phenotype data of N2 backcross animals identifies three QTL loci that contribute to the EPM phenotype. These QTL reside on chr 7, 13 and X. The chr 7 QTL is associated with resistance to EPMs, but this effect can be modified in males by an additional epistatic QTL located on the X chromosome. The interaction of these two QTL help explain the sex-specific difference observed in the N2 distribution (Figure 1a). The chr 13 QTL, on the other hand, has an effect opposite to that of the QTL at chr 7 and is associated with EPM susceptibility.

### Identification of candidate genes that influence the EPM phenotype

**Figure 3:**
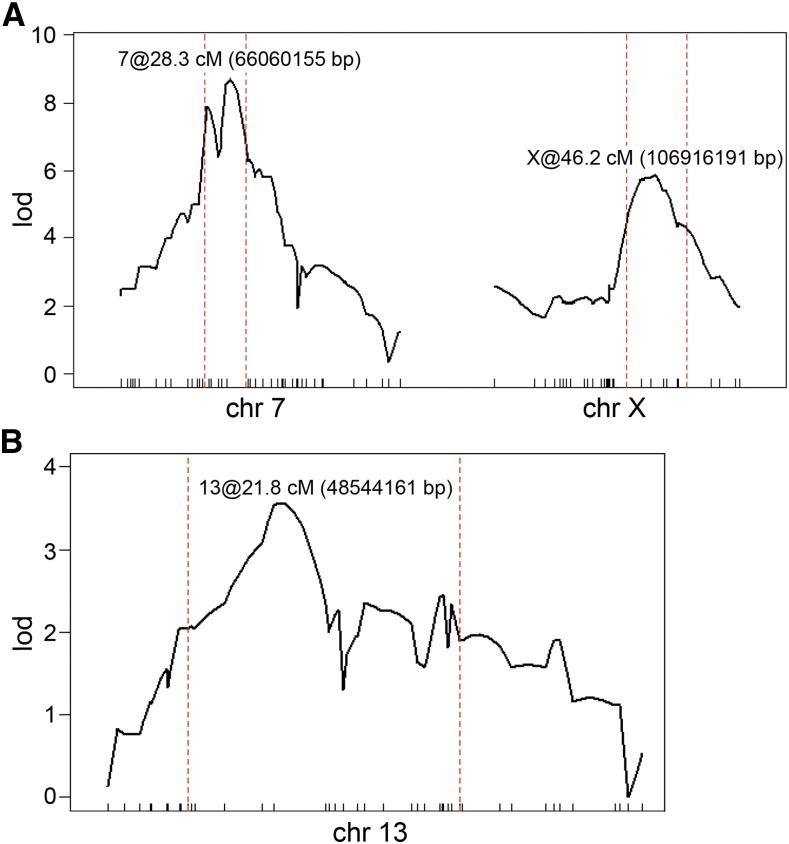
QTL linkage intervals. (A) LOD profiles at the chr 7 and X chromosome QTL after multiple-QTL analysis to fit the two QTL into one model and refine the QTL locations. (B) LOD profile at the chr 13 QTL after single-QTL analysis. 1.5-LOD linkage intervals are marked by the vertical dotted red lines. The chromosomal position (in cM and bp) with the highest LOD score is indicated for each chromosome. chr, chromosome.

As the first step to prioritizing candidate genes for the QTL identified above, we used 1.5-LOD support intervals (the chromosomal region where the LOD score is within 1.5 of its maximum) to determine the bounds of the QTL linkage region (Figure 3, Table 4). In order to consider the chr 7 and chr X QTL simultaneously we performed multiple-QTL analysis to fit these QTL into one model, refine the QTL locations, and used these LOD estimates to define the 1.5-LOD support intervals (Figure 3a). The chr 13 intervals were derived from the single-QTL analysis (Figure 3b). For these QTL, the 1.5-LOD support intervals were relatively large and encompassed a significant number of genes; the chr 7 QTL interval covered 22 Mbp with 444 genes, the chr 13 QTL covered 58 Mbp with 603 genes, and the X chromosome QTL interval covered 44 Mbps with 377 genes.

**Table 4:** 1.5-LOD support intervals for the QTL identified by single-QTL and two-QTL analysis^4^

chr	Marker (nearest SNP)	pos (cM)	pos (bp)	lod
7	c7.loc22 (CEL-7_36725559)	22.255	49625007	6.59
7	c7.loc29 (rs6160140)	28.255	66060155	8.57
7	c7.loc33 (rs3676254)	33.255	71221998	6.98
13	rs3707097	10.799	34176778	2.05
13	c13.loc20 (rs6411274)	21.790	48544161	3.56
13	rs6316213	41.589	92107548	1.90
X	cX.loc35 (gnfX.076.619)	39.169	96113472	4.28
X	cX.loc43 (rs13483929)	46.169	106916191	5.78
X	cX.loc51 (rs3697198)	56.169	139862370	4.17

4 Coordinates of 1.5 LOD intervals by chromosome (chr) provided in centimorgan position (pos (cM)) and GRCm38 chromosomal coordinate (pos (bp)) with their associated LOD score (lod).

As an approach to stratify the genes represented in these intervals we characterized them using publicly-available gene expression data, mouse resequencing data, an online program for predicting the effects of coding variants on protein function, and a curated list of genes known to be involved in pigmentation. First, any genes that are differentially expressed in skin from DBA/1J and C57BL/6J animals highlights cell-specific or systemic changes that may impact McSC function. For instance, genes that are upregulated in DBA/1J animals may be dominant drivers of EPM resistance that promote McSC stemness over differentiation, while those that are downregulated in DBA/1J may highlight proteins responsible for heightened melanogenesis in C57BL/6J animals. Using NCBI Gene Expression Omnibus (www.ncbi.nlm.nih.gov/geo/) we identified a study that compared the transcriptomes of skin derived from 11-week-old DBA/1J and C57BL/6J animals using RNAseq. This study evaluated strain-specific effects of Imiquimod on skin, and included expression data from control subjects (two males and two females for each strain) that were treated only with a non-toxic lanolin-derived occlusion cream (Swindell *et al.* 2017). Using DESeq2 to compare these DBA/1J and C57BL/6J control subjects we find a number of genes that exhibit a significant twofold difference in expression (p-adjusted < 0.05; Supplemental File 2).

Second, we were also interested to capture genes that are expressed at detectable levels in McSCs that are not up or downregulated in DBA/1J and C57BL/6J skins. We anticipate these genes represent candidates that may alter intrinsic McSC dynamics at the protein level, either through differential transcript expression or as the consequence of genetic variation in their coding sequence. Using a previously published RNAseq dataset (Harris *et al.* 2018), we generated a list of expression values from C57BL/6J wildtype McSCs isolated from dormant (telogen-stage) hairs. These gene expression values represent the read counts per gene after normalization using the median ratio method (DESeq2). Genes that were not expressed (value = 0) were excluded and the remaining genes ranked by percent (Supplemental File 3).

Third, we evaluated the genes within the linkage intervals for those that exhibit genetic variation between C57BL/6J and DBA/1J as well as those that are known to participate in pigmentation. Mouse resequencing and associated variant call data were used to identify genes that contain coding variants between C57BL/6J and DBA/1J (Mouse Genomes Project, Sanger; GRCm38)(Yalcin *et al.* 2011; Keane *et al.* 2011). These include missense, insertion and deletion mutations that are predicted to have deleterious protein consequences by PROVEAN (Choi and Chan 2015) as well as nonsense and frameshift mutations (Supplemental File 4). Genes known to be involved in pigmentation were derived from a comprehensive and curated gene list (Baxter *et al.* 2018)

Using the four criteria described above, we developed a short list of potential candidate genes. This short list includes any genes that exhibit a statistically-significant, twofold difference in gene expression between DBA/1J and C57BL/6J skins, any genes with a percent rank of expression greater than 50% in C57BL/6J McSCs that also contain a deleterious coding mutation, and any known pigmentation genes. While this approach may selectively filter out some candidate genes that are expressed nonautonomous to the McSC and those genes that are under the influence of differential transcript regulation or post-translational modifications, this abridged list provides a reasonable starting point for future follow-up (Table 5).

**Table 5:** Candidate genes for the QTL on chr 7, 13 and X ^5^

CHR	ENS GENE ID	TX START	GENE SYMBOL	DBA/B6 BASE MEAN	DBA/B6 LOG2FC	DBA/B6 PADJ	B6 McSC BASE MEAN	B6 McSC %RANK	DELETERIOUS MUTATION (PROVEAN)	PIGMENT GENE
7	ENSMUSG00000030500	51622005	Slc17a6	0.41	0.85	NA	NA	NA		yes
7	ENSMUSG00000030450	56239759	Oca2	0.76	−2.02	NA	86.28	57.1		yes
7	ENSMUSG00000025324	58658245	Atp10a	355.93	−0.34	0.3831	921.66	74.9	missense	
7	ENSMUSG00000100826	59307923	Snhg14	48.79	**1.20**	**0.0485**	201.43	62.2		
7	ENSMUSG00000070526	62461870	Peg12	23.50	**-1.17**	**0.0427**	39.34	52.4		
7	ENSMUSG00000033510	63444750	Otud7a	31.31	**1.97**	**0.0004**	14.26	45		
7	ENSMUSG00000030523	64153834	Trpm1	31.66	−0.79	0.2737	9110.73	97.5	missense, frameshift	yes
7	ENSMUSG00000030519	64501705	Apba2	74.38	**1.48**	**0.0118**	478.52	68.5		
7	ENSMUSG00000030516	65296164	Tjp1	3137.77	0.32	0.2594	8348.55	97.2		yes
7	ENSMUSG00000030554	67730159	Synm	4872.29	−0.14	0.6855	4258.52	92.6	missense	
7	ENSMUSG00000005533	67952826	Igf1r	1615.04	−0.06	0.8773	7788.35	96.9	insertion	
13	ENSMUSG00000038982	38602708	Bloc1s5	498.19	−0.08	0.8225	552.71	69.7		yes
13	ENSMUSG00000047094	40001881	Ofcc1	20.68	**-1.40**	**0.0260**	11.42	43.3		
13	ENSMUSG00000021359	40715674	Tfap2a	2043.05	0.18	0.6246	2590.07	87.4		yes
13	ENSMUSG00000021367	42301269	Edn1	315.69	0.39	0.4820	331.90	65.6		yes
13	ENSMUSG00000057531	44922078	Dtnbp1	1142.13	0.04	0.9163	1226.71	78.2		yes
13	ENSMUSG00000021375	46749086	Kif13a	2042.58	−0.23	0.4269	6899.94	96.3		yes
13	ENSMUSG00000021385	49421310	Ippk	553.23	0.40	0.2038	695.89	71.8		yes
13	ENSMUSG00000025876	54949410	Unc5a	70.35	**-2.55**	**0.0000**	76.41	56.3		
13	ENSMUSG00000025878	55027879	Uimc1	971.76	0.11	0.7791	3470.11	90.5	missense	
13	ENSMUSG00000005320	55152639	Fgfr4	231.28	**-1.08**	**0.0358**	5089.12	94.2		
13	ENSMUSG00000074885	56362900	Gm10782	9.93	**-2.74**	**0.0106**	1.02	17.8	missense	
13	ENSMUSG00000021509	56438354	Slc25a48	221.79	**1.23**	**0.0054**	92.77	57.6		
13	ENSMUSG00000097242	63289600	Gm16907	53.74	**-1.39**	**0.0320**	1001.33	75.8		
13	ENSMUSG00000021461	63304708	Fancc	276.86	**-1.10**	**0.0000**	1547.58	81		yes
13	ENSMUSG00000021466	63508327	Ptch1	425.90	0.22	0.7222	4577.16	93.3		yes
13	ENSMUSG00000021514	65278813	Zfp369	123.77	**1.06**	**0.0003**	911.45	74.7		
13	ENSMUSG00000057396	67128227	Zfp759	144.19	0.46	0.3316	120.96	59.2	deletion	
13	ENSMUSG00000058900	67173206	Rsl1	84.64	−0.57	0.0920	144.65	60.2	missense	
13	ENSMUSG00000055480	67254917	Zfp458	39.51	0.60	0.3666	100.52	58.1	missense	
13	ENSMUSG00000078994	67389308	Zfp429	128.05	0.20	0.7008	158.68	60.7	missense	
13	ENSMUSG00000055560	67405721	Zfp459	103.41	−0.04	0.9526	32.06	51	frameshift	
13	ENSMUSG00000069206	67424548	Zfp874a	368.20	−0.11	0.8147	620.83	70.8	missense	
13	ENSMUSG00000021510	67617000	Zfp729a	616.94	**1.14**	**0.0001**	581.19	70.2		
13	ENSMUSG00000090659	67779753	Zfp493	38.04	−1.22	0.0732	78.06	56.4	missense, frameshift	
13	ENSMUSG00000021594	69573448	Srd5a1	1121.55	0.37	0.1708	338.59	65.8	missense	
13	ENSMUSG00000060969	71957920	Irx1	882.46	−0.46	0.1872	2065.30	84.7		yes
13	ENSMUSG00000001504	72628819	Irx2	1889.44	0.02	0.9659	4604.11	93.3		yes
13	ENSMUSG00000021611	73627000	Tert	18.14	0.35	0.7629	162.99	60.9		yes
13	ENSMUSG00000021591	75839867	Glrx	5719.60	**1.20**	**0.0020**	149.82	60.4		
13	ENSMUSG00000021589	75869536	Rhobtb3	697.60	−0.17	0.5492	1774.22	82.7	nonsense	
13	ENSMUSG00000033991	76098733	Ttc37	766.25	0.31	0.4661	1923.19	83.8	missense	
13	ENSMUSG00000005583	83504033	Mef2c	3516.98	−0.20	0.6329	3271.05	89.9		yes
X	ENSMUSG00000031214	98554276	Ophn1	583.13	−0.10	0.8114	1988.49	84.2		yes
X	ENSMUSG00000059327	99975605	Eda	255.81	−0.20	0.6797	650.87	71.2		yes
X	ENSMUSG00000079487	101274029	Med12	1154.22	0.12	0.7437	10169.50	97.9		yes
X	ENSMUSG00000051159	102247380	Cited1	7.43	−2.07	0.0982	544.11	69.6		yes
X	ENSMUSG00000033965	103697413	Slc16a2	586.42	−0.21	0.6104	1795.94	82.9		yes
X	ENSMUSG00000033792	106027275	Atp7a	1791.99	0.61	0.0044	2306.47	86		yes
X	ENSMUSG00000033777	106143228	Tlr13	265.61	**1.39**	**0.0040**	11.25	43.1		
X	ENSMUSG00000047686	106837081	Zcchc5	66.39	**-1.04**	**0.0201**	8.77	41		
X	ENSMUSG00000079428	136224040	Tceal7	172.12	**-1.71**	**0.0000**	NA	NA		
X	ENSMUSG00000057439	136448106	Kir3dl2	29.28	**2.20**	**0.0001**	0.35	6.6		
X	ENSMUSG00000044550	136666374	Tceal3	45.61	**-1.19**	**0.0050**	152.16	60.5		
X	ENSMUSG00000087368	136742975	BC065397	23.56	−0.60	0.4724	419.10	67.3	frameshift	
X	ENSMUSG00000089768	136974021	Tmsb15b1	18.58	**-2.30**	**0.0015**	1.69	23.9	missense	
X	ENSMUSG00000042515	139210041	Mum1l1	32.08	0.04	0.9784	168.31	61.1	missense	

5 Candidate gene list for the QTL on chr 7, 13, and the X chromosome. The DBA/B6 columns refer to differential expression from RNAseq data comparing C57BL/6J and DBA/1J whole skin. Those values in bold represent genes that have both an adjusted p-value of <0.05 and a twofold change in expression in either direction. The B6 McSC columns refer to the ranked RNAseq data from C57BL/6J McSCs. Deleterious mutations were determined using PROVEAN, and the type of mutation is provided. Known pigment genes are marked with ‘yes’. ENS gene ID, Ensembl gene ID; TX START, bp position of the transcription start site (GRCm38); BASE MEAN, mean of the normalized RNAseq counts; LOG2FC, log2 fold change; PADJ, adjusted p-value; %RANK, rank of expression for the indicated gene as a percentage of the entire genes within the dataset.

## Discussion

Modifier genes are recognized for their contribution to phenotypic variation observed in disease and it’s been suggested that their identification may help predict “*sensitive pathways and nodes for therapeutic intervention* (Hamilton and Yu 2012).” With this in mind, we were interested in identifying naturally-occurring genetic variants in mouse that could contribute to variability in the phenotype of hair graying. Hair graying is the consequence of disrupting the regenerative activity of McSCs within the hair follicle or the function of their progeny. We developed a sensitized screen using mouse inbred lines as an unbiased approach to identify novel genes that participate in these mechanisms. Using the Tg(DctSox10) transgene as a condition to predispose mice to hair graying via the mechanism of McSC differentiation, we found that the DBA/1J genetic background provides resistance to this cellular phenotype (Harris *et al.* 2015). In search of modifier genes that could mitigate McSC differentiation we performed QTL analysis on progeny derived from backcrossing (C57BL/6J x DBA/1J)F1-Tg(DctSox10)/0 animals to C57BL/6J. In brief, we identified three linkage regions across three chromosomes: chr 7, 13 and the X chromosome.

While we were particularly interested in identifying QTL that could explain reduced McSC differentiation (the low EPM phenotype) associated with heterozygosity for DBA/1J, the chr 7 and 13 QTL have opposing effects consistent with promoting EPM resistance and EPM susceptibility, respectively (Figure 2a, b). Two-QTL analysis also revealed a novel, epistatic interaction between the QTL on chr 7 and the X chromosome. The effect of the X chromosome QTL is only observed in males and functions to toggle the effects of the chr 7 QTL from one of EPM resistance to EPM susceptibly when the allele at the X chromosome QTL is C57BL/6J-derived (Figure 2c). This unique interaction helps to explain the reduced effects of the DBA/1J allele at the chr 7 QTL in males (Figure 2a), as well as sex-specific skewing of the EPM phenotype in the N2 population as a whole (Figure 1).

Likely due to the relatively small number of N2 progeny evaluated for linkage, the 1.5-LOD intervals for these QTL were large and encompassed a number of genes. These were selectively filtered to include only those with differential gene expression in whole skin between C57BL/6J and DBA/1J, those with evidence to support expression within McSCs but with altered protein function, and genes associated with pigmentation phenotypes (Table 5). In considering the function of these candidate genes, we anticipated that all three of the QTL could influence McSC behavior at any number of timepoints, and include both autonomous and non-autonomous mechanisms. In general, McSCs reside in a specialized niche within the hair follicle and are activated in coordination with hair follicle stem cells. At each new hair cycle McSCs proliferate and produce progeny that will colonize the hair bulb. These progeny cells will differentiate into melanocytes and initiate the process of melanogenesis, which includes the synthesis of melanin within melanosome and the trafficking of these melanosomes for deposition into keratinocytes of the growing hair shaft (Osawa 2009). EPMs, like those in Tg(DctSox10) mice, are generated when McSCs within the stem cell niche do not self-renew properly and instead differentiate prematurely (Nishimura *et al.* 2005; Harris *et al.* 2013). Thus, resistance or susceptibility to EPMs could be the result of mitigating or exacerbating this process as early as when McSCs are deciding their fate or as late as the final steps of pigment production. In addition, because the initial EPM phenotype is driven by the Tg(DctSox10) transgene as a consequence of *Sox10* overexpression, these QTL could also act as regulators of SOX10.

As one example, there are a number of genes within the linkage intervals for chr 7, 13 and the X chromosome that have known roles in cellular mechanisms associated with the function of the melanosome organelle. These include *Oca2*, *Trpm1*, *Bloc1s5*, *Dtnbp1*, *Kif13a*, and *Atp7a*. Since our phenotypic assessment of EPMs is dependent on the production of visual pigmentation, variability in the EPM phenotype may reflect changes in melanosome biogenesis or maturation. In particular, *Trpm1* encodes a protein called transient receptor potential cation channel, subfamily M, member 1 (also known as melastatin) and is highly expressed in C57BL/6J McSCs (Table 5). TRPM1 localizes to non-melanosomal vesicles and its activity is associated with increased intracellular melanin content (Oancea *et al.* 2009). Analysis of mouse resequencing data demonstrates that the *Trpm1* gene contains DBA/1J-related missense and frameshift coding mutations that are predicted to alter protein function (Table 5). Thus it follows that the low EPM phenotype associated with the DBA/1J allele at the chr 7 QTL could be a consequence of malfunctional TRPM1 protein, and a subsequent reduction in pigment production in Tg(DctSox10) McSCs. Interestingly, *Trpm1* knockout in mouse produces no apparent congenital pigmentation defect on its own (Koike *et al.* 2010) but is consistent with our hypothesis that, as a modifier gene, it may only act to modify previously existing coat color phenotypes in mouse.

As a second example, quite a few of the candidates identified are involved in transcriptional regulation and could play a role in shaping McSC self-renewal or differentiation. These include *Irx1*, *Irx2*, *Mef2c*, *Rsl1*, *Tfap2a*, *Zfp369*, *Cited1* and *Med12*. *Mef2c* and *Tfap2a* are contained within the chr 13 QTL interval, are expressed at relatively high levels in C57BL/6J McSCs (Table 5), and both have known function in promoting melanocyte differentiation (Agarwal *et al.* 2011; Seberg *et al.* 2017). The DBA/1J allele at the chr 13 QTL is associated with EPM susceptibility making *Mef2c* and *Tfap2a* plausible candidates for enhancing premature differentiation of Tg(DctSox10) McSCs.

As this study highlights, one benefit to using the natural variation of inbred mouse lines to evaluate QTL is that many of the lines have now been sequenced (Keane *et al.* 2011). In addition, the structural variation between these lines and the reference genome is known and searchable (Yalcin *et al.* 2011). While additional fine mapping or the use of chromosome substitution mouse lines are helpful to shrink large linkage intervals, genomic tools, like computational prediction of protein function and whole genome gene expression data are now more readily available and can be applied to the identification of promising candidates. This is particularly helpful when limited resources prohibit the generation of additional crosses to refine the linkage region. With this approach we identified a list of gene candidates that may contribute to DBA/1J-specific changes in McSC differentiation and each will have to be tested individually for causality. DBA/1J and DBA/2J share sufficient homology that BAC transgenesis using available DBA/2J BAC clones (MM_DBa) may be a good way to begin confirming these candidates. For those genes that contain missense mutations of varying predicted consequence, assessing each for its effects on protein secondary structure *in silico* (*e.g.*, using the RCSB Protein Data Bank), and its effects on stability and subcellular localization *in vitro* can help in the selection of those that are more likely to be detrimental to cell function *in vivo*. In addition, EPM phenotyping has been performed on other genetic backgrounds (Harris *et al.* 2015), and any variants that are common between strains that exhibit the low EPM phenotype and distinct between the high EPM strains could provide additional support for specific candidates. Finally, with the versatility of CRISPR/Cas9-mediated genome editing, creating mice with specific candidate gene defects will help to finalize the identification of genes involved in the variation of McSC differentiation, a cellular pathology associated with hair graying.
